# The Essential Nucleolar Yeast Protein Nop8p Controls the Exosome Function during 60S Ribosomal Subunit Maturation

**DOI:** 10.1371/journal.pone.0021686

**Published:** 2011-06-29

**Authors:** Marcia C. T. Santos, Mauricio B. Goldfeder, Nilson I. T. Zanchin, Carla C. Oliveira

**Affiliations:** 1 Department of Biochemistry, Institute of Chemistry, University of São Paulo, São Paulo, Brazil; 2 Centro de Biologia Molecular e Engenharia Genética, Universidade Estadual de Campinas, São Paulo, Brazil; 3 Faculdade de Ciências Aplicadas, Universidade Estadual de Campinas, Limeira, São Paulo, Brazil; University of Medicine and Dentistry of New Jersey, United States of America

## Abstract

The yeast nucleolar protein Nop8p has previously been shown to interact with Nip7p and to be required for 60S ribosomal subunit formation. Although depletion of Nop8p in yeast cells leads to premature degradation of rRNAs, the biochemical mechanism responsible for this phenotype is still not known. In this work, we show that the Nop8p amino-terminal region mediates interaction with the 5.8S rRNA, while its carboxyl-terminal portion interacts with Nip7p and can partially complement the growth defect of the conditional mutant strain *Δnop8/GAL::NOP8*. Interestingly, Nop8p mediates association of Nip7p to pre-ribosomal particles. Nop8p also interacts with the exosome subunit Rrp6p and inhibits the complex activity *in vitro*, suggesting that the decrease in 60S ribosomal subunit levels detected upon depletion of Nop8p may result from degradation of pre-rRNAs by the exosome. These results strongly indicate that Nop8p may control the exosome function during pre-rRNA processing.

## Introduction

Eukaryotic ribosome maturation is a complex pathway that requires at least 200 transacting factors in addition to the ribosomal protein subunits (r-proteins) and the ribosomal RNAs (rRNAs). In yeast, three rRNAs (18S, 5.8S and 25S) are transcribed as a 35S precursor by RNA polymerase I, while RNA polymerase III transcribes 5S rRNA. 35S pre-rRNA processing involves several endo- and exonucleolytic cleavage reactions, as well as nucleotide modifications at specific positions. Pre-rRNA processing starts co-transcriptionally in the nucleolus, continues through the nucleoplasm, and the final reactions take place in the cytoplasm [1].

Throughout pre-rRNA processing and assembly of r-proteins, precursor ribosome particles are formed, that are going to originate the 40S and 60S mature ribosome subunits. The initial 90S particle undergoes cleavage at site A_2_, originating the pre-40S and pre-60S. A large number of proteins have been characterized to participate in the pre-ribosomal particles during their maturation, many of which are transiently associated with the 90S, pre-60S and pre-40S particles and are known as pre-ribosomal proteins. While in the nucleolus, associated factors participate in the pre-60S subunit processing reactions for pre-rRNA 27S maturation. During transport of pre-60S ribosomes to the nucleoplasm, the protein composition of the particles undergoes great changes, by removal of some factors and joining of others. Similar changes occur during transport of pre-60S from the nucleoplasm to the cytoplasm, where final rearrangements will give rise to mature 60S ribosomal subunits [2].

Nop8p was first identified as a Nip7p interacting partner required for 60S ribosome subunit biogenesis [3], and was later shown to genetically interact with the predominantly nucleolar RNA helicase Dbp6p, also involved in 60S biogenesis [4]. Many helicases have been shown to be necessary for pre-rRNA processing and ribosome assembly, participating in molecular rearrangements during these processes. Interestingly, some helicases are specific for pre-40S or pre-60S maturation. DDX51 has recently been shown to be required for the release of U8 snoRNA from pre-rRNA 46S, necessary for the formation of the 28S rRNA mature 3′-end in mammalian cells [5]. The Nop8p human homolog, NOP132, has been shown to interact with the DEAD-box RNA helicase DDX47, also involved in pre-rRNA processing [6].

The pre-60S subunit particle has been isolated with the nuclear GTPase Nug1p, in which a large number of non-ribosomal proteins were identified [7]. Purification of intermediate pre-60S complexes with various processing factors has also provided information on the order of binding and composition of assembly intermediates [8]. Nop8p has been identified in pre-60S complexes purified with different tagged proteins. It has been shown to be associated with the nucleolar protein TAP-Npa1p [9], and with TAP-Npa2p in a subcomplex formed by Dbp6p, Npa1p (Urb1p), and Rsa3p [10]. Interestingly, purification of the pre-60S complex with processing factors has not resulted in the identification of ribonucleases, such as the exosome, Rat1p and Xrn1p, which function in maturation of 60S rRNAs and might associate transiently with the processing complex [11].

The exosome is required for many RNA maturation and degradation activities both in the nucleus and cytoplasm. In yeast, the nuclear and cytoplasmic complexes share all but one subunit, Rrp6p, which along with Rrp44p is responsible for the catalytic activity of the nuclear complexes [12]. The nuclear exosome is required for maturation of the 3′-end of the 5.8S rRNA and for degradation of the 5′-ETS spacer sequence [13]–[15]. Many factors interact with the yeast exosome, directing the complex to different substrates and affecting its activity [16], [17]. In a previous study, we have shown that the yeast nucleolar protein Nop53p stimulates the exosome *in vivo* during 5.8S rRNA maturation and *in vitro* for degradation of RNA oligonucleotides [18], [19]. Here we show that yeast Nop8p plays a role opposite to Nop53p, inhibiting the exosome *in vitro*, providing the first example of a eukaryotic protein controlling the RNase activity of the exosome.

## Results

### The C-terminal portion of Nop8p is essential for its function

**Figure 1:**
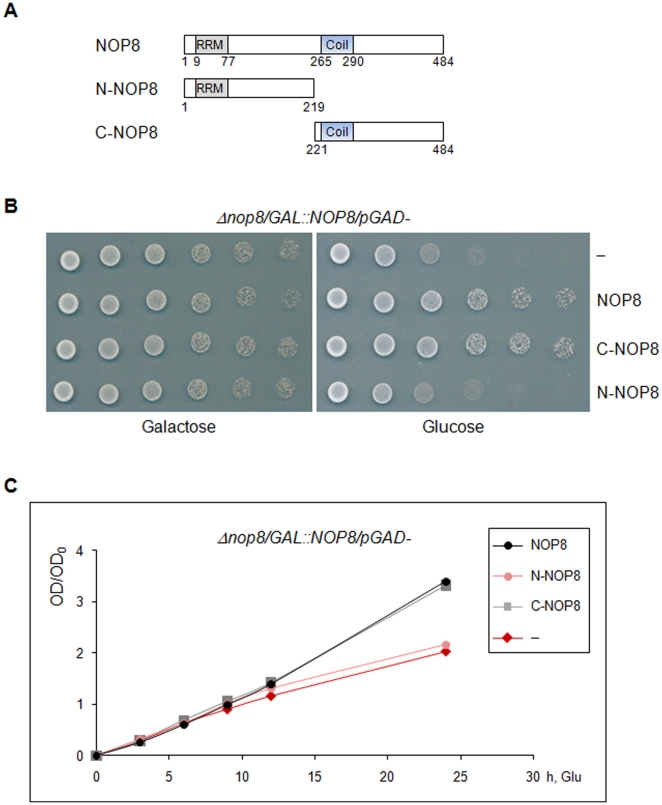
Complementation analysis of *Δnop8/GAL::NOP8* strain by the truncated mutants of Nop8p, N-Nop8p and C-Nop8p. (**A**) Diagram representing Nop8p truncated mutants. (**B**) Serial dilutions of *Δnop8/GAL::NOP8* cells expressing either full-length or mutants of Nop8p under the control of a constitutive promoter were plated on galactose- or glucose-containing media. C-Nop8p rescues growth in glucose medium. (**C**) Growth curve of the same strains growing in glucose-containing medium.

Nop8p is a 57 kDa essential yeast protein that contains a putative RNA binding domain in its N-terminal portion, a coiled-coil region in its C-terminal portion, and has previously been shown to localize to the nucleolus, to interact with Nip7p, and to be involved in pre-rRNA processing [3]. To understand how Nop8p acts during ribosome biogenesis, we explored the contribution of its amino- and carboxyl-portions to its function. Therefore, deletion mutants of Nop8p were cloned under the control of a constitutive promoter and expressed in the conditional strain *Δnop8/GAL1::NOP8*, so that expression of full-length Nop8p could be downregulated in the presence of glucose. The complementation analysis shows that the C-terminal portion of Nop8p, ranging from amino acid residue 221 through 484, is sufficient for rescuing growth of the conditional strain in glucose medium, whereas the N-terminal portion of the protein (amino acid residues 1 through 219) does not show any complementation effect (Fig. 1).

**Figure 2:**
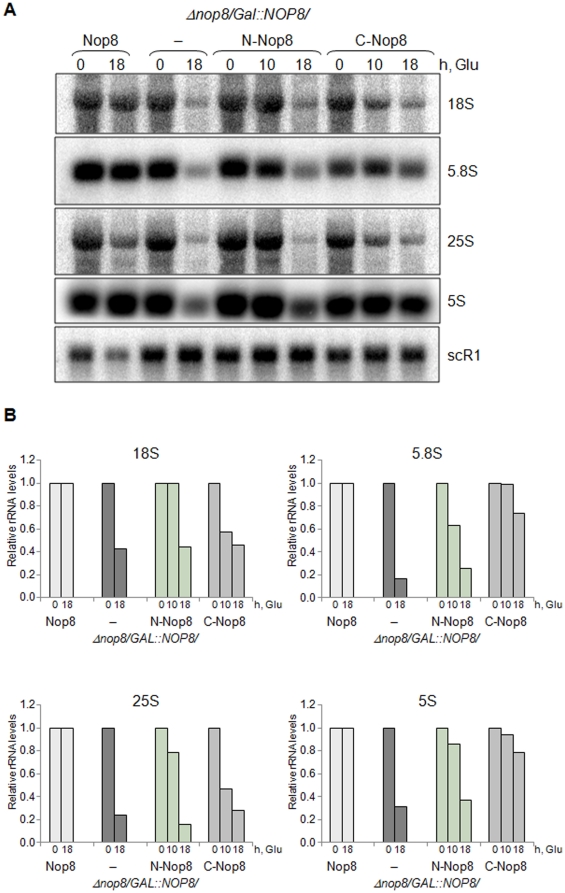
Analysis of rRNA levels in strains expressing Nop8p truncated mutants by northern hybridization. (**A**) Total RNA was extracted from cells growing in galactose (t_0_) or in glucose-containing medium for 10 or 18 hours and subjected to northern hybridization with probes specific for mature rRNAs. (**B**) Relative rRNA levels were obtained after quantitation of the bands in a Phosphorimager. Values correspond to rRNA levels corrected for the scR1 RNA; t/t_0_.

Northern blot analysis of rRNAs steady-state levels shows that depletion of Nop8p leads to a decrease in all mature rRNAs levels, but it affects more severely the 60S subunit rRNAs 5.8S, 25S, and 5S. Similar to the conditional strain *Δnop8/GAL1::NOP8* depleted of Nop8p (in glucose medium), expression of the N-Nop8p deletion mutant leads to decreased levels of mature rRNAs. Expression of C-Nop8p, however, restores levels of the mature 5.8S and 5S rRNAs, although the levels of the 18S and 25S rRNAs are only slightly higher in this strain than in the conditional mutant (Fig. 2).

**Figure 3:**
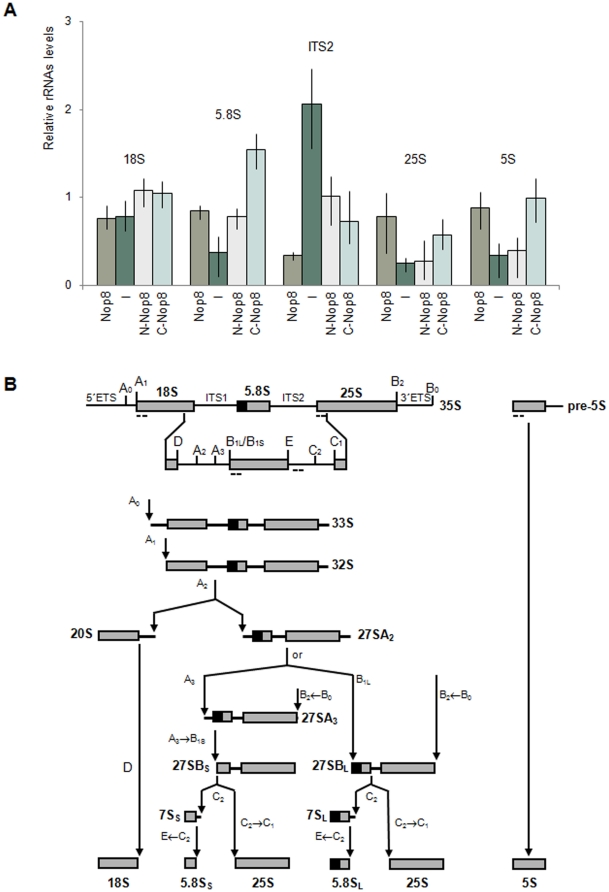
Analysis of rRNA levels in strains expressing truncated Nop8p mutants by qPCR. (**A**) qPCR was performed with total RNA extracted from conditional strain *Δnop8/GAL::NOP8* expressing Nop8p or its truncated versions using primers complementary to different regions of the rRNAs. Relative positions of the primers are shown in **B**. Quantitation was performed as described previously (Livak and Schmittgen, 2001). Results from nine different experiments are expressed as means fold change ± S.D. (**B**) Pre-rRNA processing pathway in *Saccharomyces cerevisiae*. The 35S pre-rRNA contains sequences for mature 18S, 5.8S, and 25S rRNAs (represented as grey boxes) along with additional internal and external spacer sequences (represented as horizontal lines). The 35S pre-rRNA is transcribed by RNA polymerase I and rapidly modified and processed to produce the 33S pre-rRNA. Cleavage of 33S pre-rRNA at site A_0_ generates the 32S pre-rRNA. The 20S and 27SA_2_ pre-rRNA processing intermediates are generated through internal cleavage of 32S pre-rRNA at the A_2_ site. Subsequent processing and cleavage of 20S and 27SA_2_ pre-rRNAs result in the production of the mature 18S, 25S, and 5.8S rRNAs, respectively. The 5S rRNA is transcribed separately by RNA polymerase III.

In addition to the northern hybridization experiments, the effect of the expression of the Nop8p deletion mutants on rRNAs levels was analyzed by qPCR. In these assays, small portions of the rRNAs were amplified with specific primers after the synthesis of cDNA with hexarandom primers. The results show that the Nop8p depletion causes a severe decrease in the levels of the mature rRNAs of the 60S ribosomal subunit and a concomitant increase in the levels of pre-rRNAs containing the ITS2 region (Fig. 3), in accordance with the northern hybridizations and previous analyses (Fig. 2; [3]). Expression of N-Nop8p does not restore 25S rRNA levels, although it causes a decrease in the levels of pre-rRNAs containing the ITS2 region. C-Nop8p, on the other hand, restores levels of most mature rRNAs, and particularly of the 5.8S and 5S rRNAs, while decreasing the levels of pre-rRNAs containing the ITS2 spacer sequence (Fig. 3). The primers used in the qPCR reactions are complementary to the internal ITS2 region and cannot be used to distinguish between pre-rRNAs 35S, 27S, and 7S, all of which are affected by the depletion of Nop8p. The increase in the levels of pre-rRNAs containing the ITS2 region is most probably due to the accumulation of unprocessed 35S pre-rRNA upon depletion of Nop8p [3].

**Figure 4:**
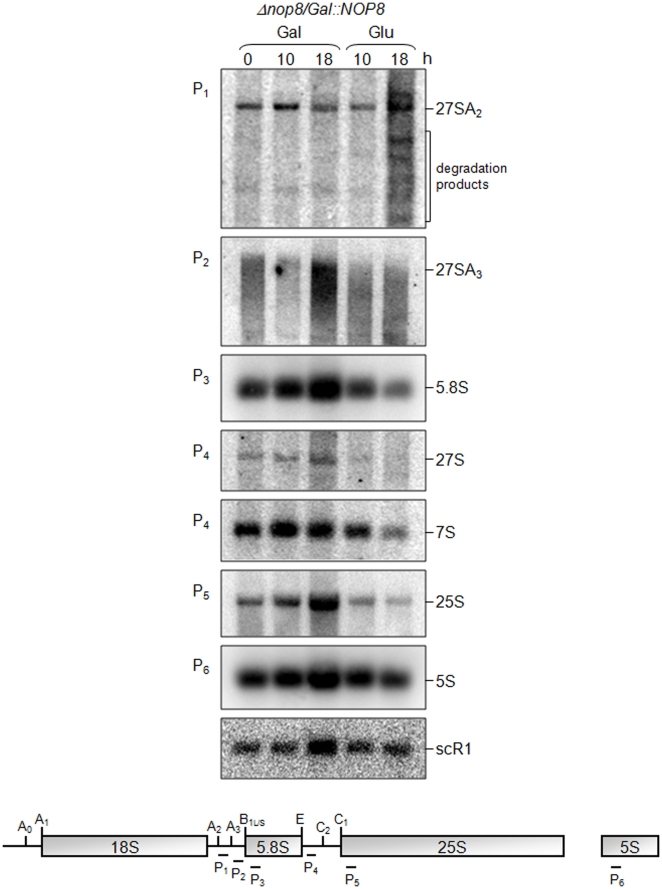
Analysis of the effect of Nop8p expression on rRNA levels in strain *Δnop8/GAL1::NOP8* by northern hybridization. Total RNA was extracted from cells growing in galactose (t_0_) or in glucose-containing medium for 10 or 18 hours and subjected to northern hybridization with probes specific for precursors or mature rRNAs. Relative positions of the probes used in the hybridizations are shown on the bottom.

Due to the stronger effect of the Nop8p depletion on the 60S ribosomal subunit RNAs, additional northern hybridizations were performed to analyze the 5′-ends of the 27S pre-rRNAs (Fig. 4). In these assays, it was possible to distinguish between the 27S pre-rRNA species, and to conclude that in the absence of Nop8p the 27SA_2_ precursor rRNA is subjected to alternative processing pathways, generating products visualized as shorter bands (Fig. 4; probe P_1_). Interestingly, similar phenotypes have been reported for the proteins involved in 60S maturation, Npa1p and Npa2p, that also interact with Nop8p [9], [10], [20]. In addition, these results show that the 27SA_3_ pre-rRNA is directed for degradation upon depletion of Nop8p, visualized as a smear on the northern blot (Fig. 4; probe P_2_). Consequently, the levels of the 27SB pre-rRNA and the mature rRNAs 25S and 5.8S decrease over time upon Nop8p depletion (Fig. 4).

The effects of the expression of the Nop8p deletion mutants on 60S ribosomal subunit maturation were further analyzed by sucrose gradient fractionation. The results show that in the absence of Nop8p, the 60S ribosomal subunit levels are significantly lower than in the presence of Nop8p (Fig. S1, upper panels). In agreement with the results presented above, in the strain expressing C-Nop8p, the 60S levels are similar to those of the strain expressing full-length Nop8p, whereas expression of N-Nop8p does not restore 60S levels (Fig. S1, lower panels).

**Figure 5:**
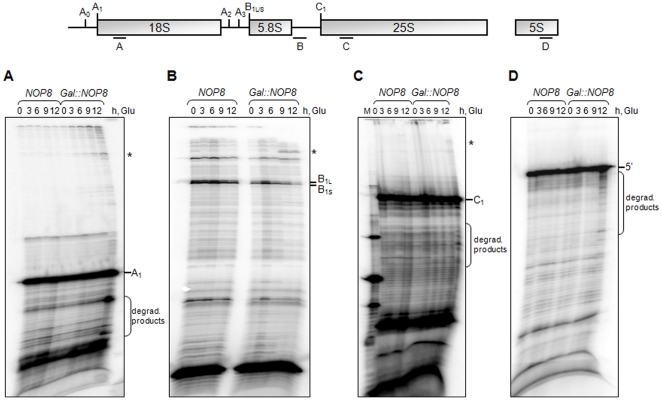
Processing of 35S pre-rRNA was analyzed by primer extension reactions of total RNA extracted from cells growing in media containing either galactose (0 h) or glucose (up to 12 h). Relative positions of the primers used in the primer extension reactions are shown on top. (**A**) Primer extension with the primer A allows the detection of the site A_1_. Bands corresponding to precursor rRNAs, as well as degradation products are detected. (**B**) Primer extension reactions with primer B, complementary to ITS2 shows the accumulation of pre-rRNAs upon depletion of Nop8p. (**C**) Reactions with primer C that hybridizes in the 5′-end region of 25S rRNA shows the degradation products generated by the absence of Nop8p. (**D**) Reactions with primer D, complementary to the 5S rRNA show that the depletion of Nop8p leads to the degradation of this rRNA. Asterisks indicate longer extensions of the reactions corresponding to precursor RNAs.

rRNA processing was also analyzed by primer extension assays, through which it was possible to confirm that the depletion of Nop8p leads to an inhibition of pre-rRNA processing and causes precursor rRNAs to be directed for degradation (Fig. 5). Primer extension reactions with an oligonucleotide complementary to the 5′-end region of the 18S rRNA show that although the mature 5′-end of the 18S rRNA is detected 12 hours after inhibition of Nop8p expression, bands corresponding to pre-rRNAs can be visualized in addition to bands corresponding to degradation products (Fig. 5A). Reactions with a primer complementary to the ITS2 region show that 9 hours after transfer to glucose medium the bands corresponding to the mature 5.8S 5′-ends become weaker and higher bands can be visualized, confirming the strong 27S pre-rRNA processing defect caused by the depletion of Nop8p (Fig. 5B). Similarly, reactions with a primer specific for the 5′-end region of the 25S rRNA show that the depletion of Nop8p causes processing defect of 27S pre-rRNA, detected as higher bands on the gel. Bands corresponding to degradation products are also detected (Fig. 5C). Interestingly, depletion of Nop8p leads to the formation of 5S rRNA degradation products, visualized as lower bands on the gels (Fig. 5D). It is possible that the defect on pre-rRNA 27S processing and consequent decreased levels of mature rRNAs 25S and 5.8S caused by the depletion of Nop8p result in a defective assembly of 60S subunits that can lead to faster degradation of all 60S components, including the 5S rRNA. Accordingly, previous results have already shown that 5S rRNA levels are influenced by the rate of processing of the 27S pre-rRNA [21]. These results are in agreement with rRNA steady-state level analysis and show that Nop8p depletion leads to a decreased efficiency of pre-27S processing and to the degradation of pre-rRNAs.

### The N-terminal portion of Nop8p mediates association with 5.8S rRNA-containing complexes

**Figure 6:**
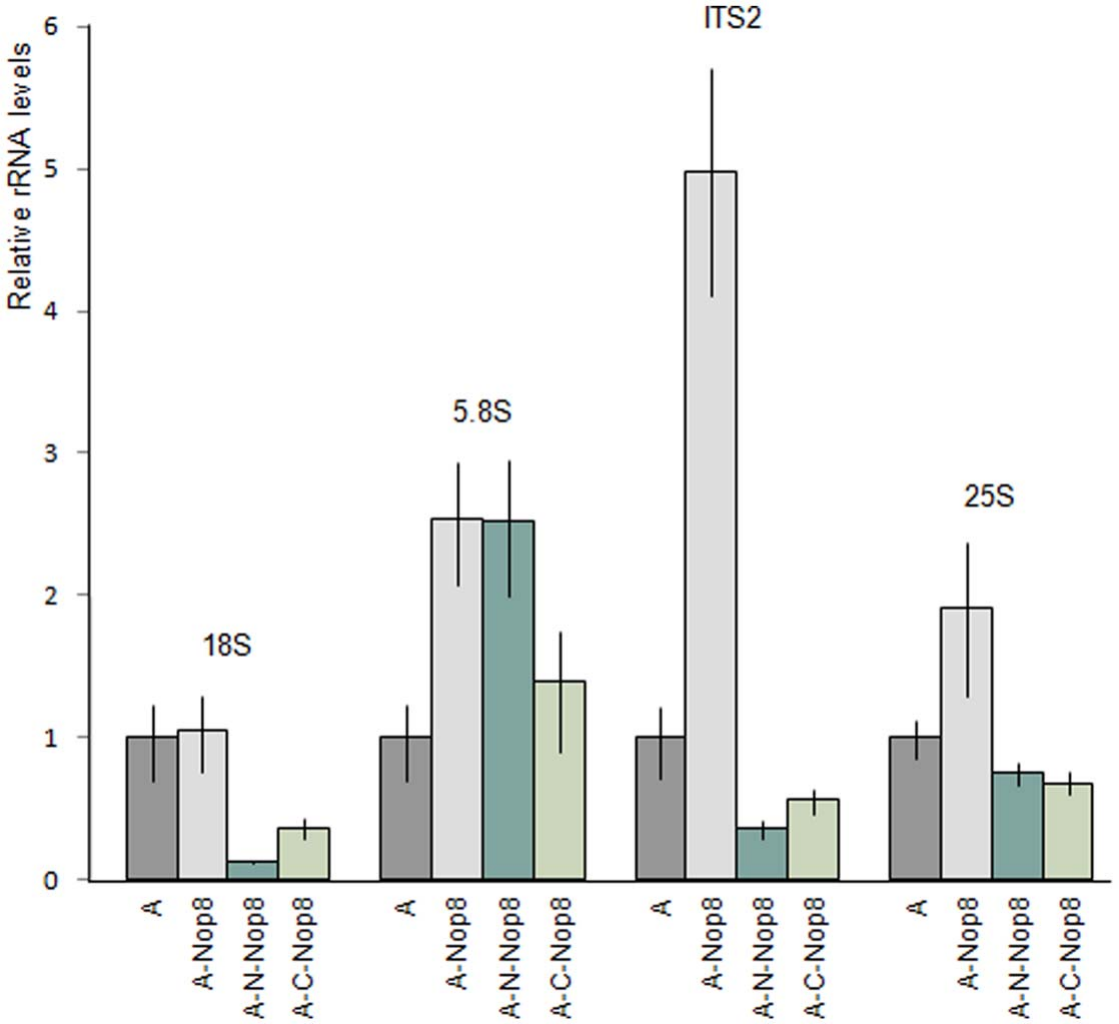
RNA co-immunoprecipitation with ProtA-Nop8p and its deletion mutants. Total extracts from cells expressing either ProtA, ProtA-Nop8p, ProtA-N-Nop8p, or ProtA-C-Nop8p were incubated with IgG-sepharose beads. After immunoprecipitation, RNA was extracted from the bound material and analyzed by qPCR with primers specific for the RNAs indicated on the top. Quantitation was performed as described previously (Livak and Schmittgen, 2001). Results from three different experiments are expressed as means fold change ± S.D.

Based on the involvement of Nop8p in pre-rRNA processing and the presence of a putative RRM at the Nop8p N-terminal portion, we carried out RNA co-immunoprecipitation assays to analyze whether Nop8p co-purifies specific rRNAs. Nop8p and the Nop8p deletion mutants were therefore fused to a protein A tag and expressed under the control of the *GAL1* promoter. The full-length Nop8p and truncated mutants were immobilized on IgG-Sepharose beads for the analysis of interacting complexes. To determine the RNA composition of the complexes, RNA was extracted from the precipitated fractions and analyzed by qPCR, corrected by the RNAs co-precipitated with protein A alone. The results show that the ProtA-Nop8p fusion co-immunoprecipitates the pre-rRNAs containing the ITS2 region, and the mature rRNAs 25S and 5.8S (Fig. 6). Interestingly, the Nop8p deletion mutant ProtA-N-Nop8p co-immunoprecipitated 5.8S rRNA as efficiently as the full-length ProtA-Nop8p, while ProtA-C-Nop8p co-immunoprecipitated only 5.8S rRNA, but not very efficiently (Fig. 6). These results suggest that Nop8p interacts with pre-60S particles through its N-terminal portion, which contains an RNA recognition motif and may bind to the 5.8S rRNA directly.

### Nop8p mediates association of Nip7p with pre-ribosomes via its C-terminal portion

**Figure 7:**
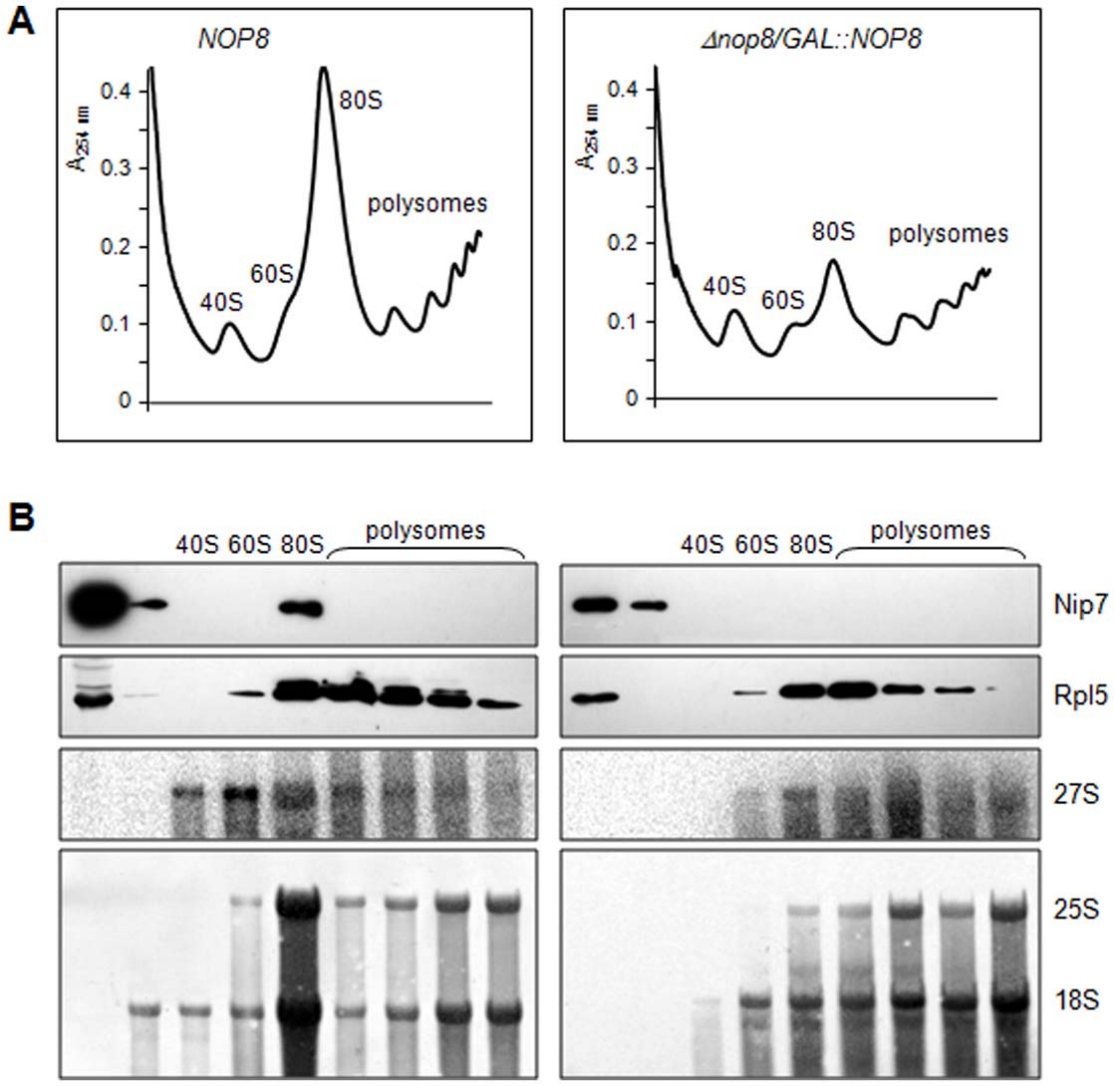
Analysis of the polysomal profiles of strain *Δnop8/GAL1::NOP8*. *NOP8* and *Δnop8/GAL1::NOP8* strains were incubated in glucose-containing medium for 6 hours for the analysis of polysomal profiles through sucrose gradient. (**A**) Left panel, *NOP8* strain. Right panel, *Δnop8/GAL1::NOP8* strain showing very low levels of the 60S ribosomal subunit, and consequent decrease in 80S ribosomes and polysomes. (**B**) Detection of Nip7p cosedimenting with pre-60S particles by Western blot of fractions from the polysomal gradients. Western blots with antiserum against Rpl5p were performed as controls. RNA was extracted from gradient fractions and analyzed by northern hybridizations. Lower levels of mature rRNAs in the strain *Δnop8/GAL1::NOP8* are due to degradation upon depletion of Nop8p.

Based on the results of Nop8p interaction with rRNAs described above, and on Nop8p interaction with Nip7p [3], we next sought to determine the association of Nip7p with pre-ribosomes in the absence of Nop8p. Polysomal profile analysis was therefore performed with extracts from *NOP8* and *Δnop8/GAL1::NOP8* cells. Interestingly, in the presence of Nop8p, Nip7p is concentrated in the fractions containing the pre-60S complex, whereas in the absence of Nop8p, endogenous Nip7p no longer associates with pre-60S particles (Fig. 7B). The ribosomal protein Rpl5p was used as a control and its sedimentation was not affected by the depletion of Nop8p (Fig. 7B). RNA extracted from the same fractions and analyzed by northern hybridization confirms that Nip7p cosediments with fractions containing 27S pre-rRNA. These results suggest that Nop8p is important for Nip7p interaction with pre-60S complex, or that association of Nip7p requires intact pre-60S. Northern hybridization of RNAs extracted from the polysomal gradient fractions show the degradation of the 27S pre-rRNA in the absence of Nop8p, which can be visualized as a smear instead of defined bands (Fig. 7B, right panels). Depletion of Nop8p has been shown to cause degradation of pre-rRNAs, leading to lower concentrations of mature rRNAs [3], which was confirmed by the primer extension reactions described above and the polysomal profile analysis. The data obtained in this work indicate that Nop8p binds to pre-60S before Nip7p and thereby stabilizes the 27S pre-rRNA, and may serve as a binding platform for Nip7p.

**Figure 8:**
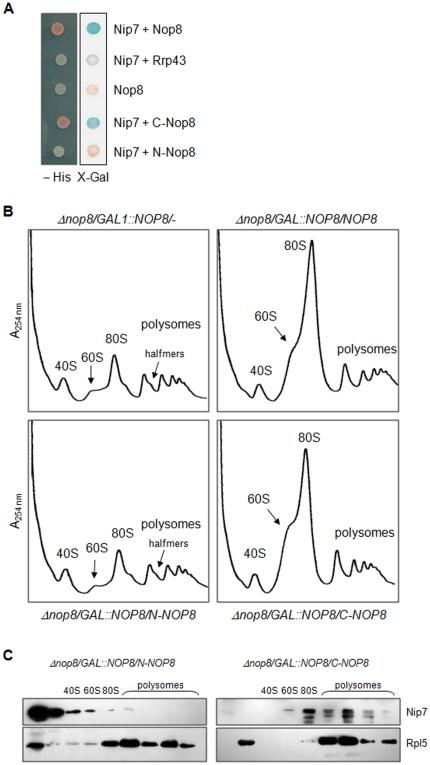
Mapping of Nop8p domain responsible for interaction with Nip7p. (**A**) The interaction between Nop8p truncation mutants and Nip7p was analyzed using the two-hybrid system. Nop8p truncation mutants were fused to Gal4p transcription activation domain (AD) and tested for interaction with Nip7p, which was fused to *lex*A DNA binding domain (BD). Expression of the reporter genes *HIS3* and *lac*Z was determined by the growth of yeast strains on plates without histidine (-His, 1 mM 3-aminotriazole), or by β-galactosidase activity assays (X-Gal). Nop8p interacts with Nip7p through its C-terminal portion. (**B**) Analysis of polysomal profiles of strain *Δnop8/GAL1::NOP8* expressing either full-length Nop8p, N-Nop8p or C-Nop8p. Nop8p was depleted by incubating cells in glucose-containing medium for 12 hours. Top left panel, control strain. Top right panel, *Δnop8/GAL1::NOP8/NOP8* strain. Bottom left panel *Δnop8/GAL1::NOP8/N-NOP8* strain. Bottom right panel, *Δnop8/GAL1::NOP8/C-NOP8*. (**C**) Analysis of Nip7p co-sedimentation with polysomes. Fractions from polysomal profiles were analyzed by Western blot for the detection of Nip7p. Western blot with antiserum against Rpl5p was performed as a control. Expression of C-Nop8p is sufficient for the Nip7p association with pre-60S particles.

To map the Nop8p region involved in the interaction with Nip7p, two-hybrid assays were performed and show that the C-terminal portion of Nop8p is sufficient for interaction with Nip7p (Fig. 8A). Further analysis of interaction between Nop8p and Nip7p were performed by determining Nip7p sedimentation on sucrose gradients in the presence of Nop8p deletion mutants. Polysomal profile analyses of extracts from cells expressing the Nop8p deletion mutants confirm that the C-terminal portion partially complements the function of this protein (Fig. 8B; lower right panel). To analyze the effect of Nop8p deletion mutants on Nip7p sedimentation on sucrose gradients, proteins extracted from the gradient fractions were subjected to western blots. Interestingly, expression of N-Nop8p is not sufficient for the association of Nip7p with pre-60S complex (Fig. 8C). The results of the expression of N-Nop8p upon Nip7p sedimentation on sucrose gradients are similar to those obtained in the absence of Nop8p (Fig. 7B), strengthening the hypothesis that Nop8p is necessary for Nip7p binding to the pre-60S particles. In the presence of C-Nop8p, on the other hand, Nip7p is associated with pre-60S and larger complexes (Fig. 8C), suggesting that the expression of C-Nop8p blocks the dissociation of Nip7p from the pre-60S complex. Similarly, the depletion of the 60S protein P0 has been shown to cause the retention of Mrt4p in pre-60S particles [22]. These results strongly suggest that the interaction between Nop8p and Nip7p through Nop8p C-terminal portion is important for Nip7p binding to pre-ribosome complexes, and that full-length Nop8p is required for the release of Nip7p from the pre-60S particles.

### Nop8p interacts with the exosome subunit Rrp6p and inhibits exosome activity

**Figure 9:**
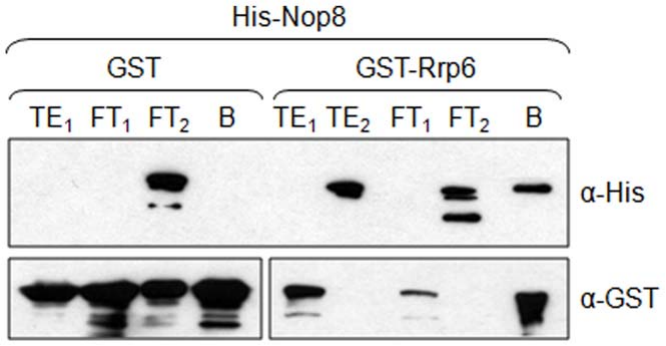
Nop8p interacts with the exosome subunit Rrp6p. Western blot for detection of proteins after pull-down assay. Total extract from cells expressing either GST or GST-Rrp6p (TE_1_) was incubated with glutathione-sepharose beads, flow through fraction was collected (FT_1_) and after washing, total extract of cells expressing His-Nop8p (TE_2_) was loaded. Flow through fraction was collected again (FT_2_), resin was washed, and bound fraction obtained (B). His-Nop8p is only pulled-down by GST-Rrp6p. His-Nop8p was detected with anti-His monoclonal antibody. GST-Rrp6p and GST were detected with anti-GST anti-serum.

As shown here and in a previous study [3], depletion of Nop8p leads to the degradation of pre-rRNAs. Since the exosome complex is involved in quality control of rRNAs [12], [17], we next investigated whether Nop8p could interact with any of the exosome subunits. Initial experiments to detect possible interactions were performed by using the two-hybrid system which indicated an interaction between Nop8p and Rrp6p (data not shown). Subsequent protein pull-down experiments confirmed the direct interaction between recombinant His-Nop8p and GST-Rrp6p (Fig. 9). Nop8p contains large regions that are predicted to be natively disordered. Intrinsically disordered regions have higher flexibility and provide also larger binding interfaces when compared to folded proteins of the same size, allowing them to fit a variety of different binding partners [23]. Therefore, the size and flexible structure of Nop8p provides space for the interaction with RNA at the N-terminal portion and with different partners in the C-terminal portion.

To determine whether Nop8p could affect the exosome RNase activity *in vitro*, we next purified the exosome complex with TAP-Rrp43p, as described previously [19]. To ascertain that the bound complex corresponded to the exosome complex, immunoblot was performed with antiserum against another exosome subunit, Mtr3p (Fig. S2A). As a negative control for the *in vitro* RNase activity assays, box C/D snoRNPs were purified by the same procedure with TAP-Nop58p [19]. The TAP-Nop58p bound complex was tested for the presence of another box C/D snoRNP subunit, Nop1p, confirming its presence (Fig. S2B).

**Figure 10:**
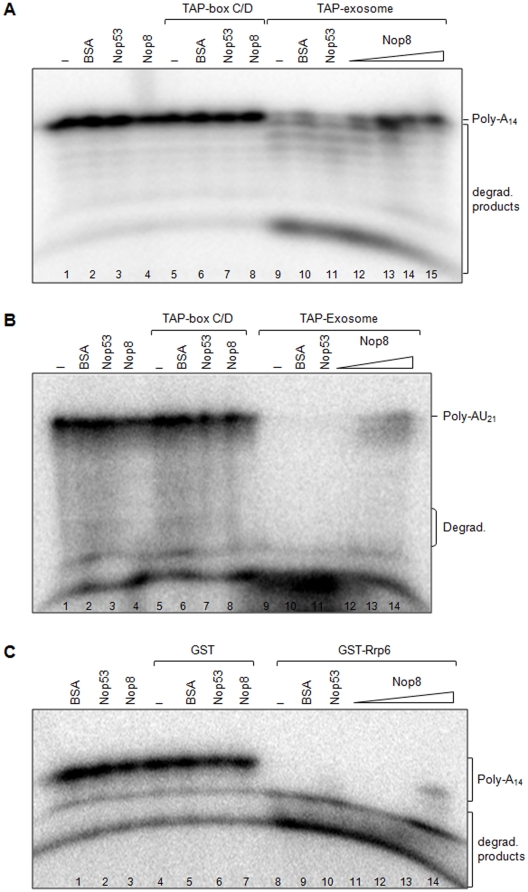
*In vitro* RNA degradation assay to test the effect of Nop8p on the exosome activity. (**A**) Radioactively labeled poly-A_14_ RNA oligo was incubated with 2 µM of the exosome complex isolated with TAP-Rrp43p, or 5 µM of box C/D snoRNP isolated with TAP-Nop58p, and 1.5, 5, 10 or 15 pmol of His-Nop8p, 15 pmol of His-Nop53p or 15 pmol of BSA. Reactions were run for 30 min at 37°C and the products were analyzed by denaturing polyacrylamide gel electrophoresis. The main degradation products generated by the exosome complex are indicated. This is a representative figure of five independent experiments with different protein preparations. (**B**) Radioactively labeled poly-AU_21_ RNA oligo was incubated with 2 µM of the exosome complex isolated with TAP-Rrp43p, or 5 µM of box C/D snoRNP isolated with TAP-Nop58p, and 30 pmol of His-Nop53p or 30 pmol of BSA, or increasing amounts of His-Nop8p (1 to 30 pmol of His-Nop8p). (**C**) Radioactively labeled poly-A_14_ RNA oligonucleotides were incubated with 100 pmol of either GST or GST-Rrp6p, in the presence of 5, 10, 15 or 25 pmol of His-Nop8p, 25 pmol His-Nop53p or 25 pmol BSA, as described in **A**.

*In vitro* RNA degradation assays were performed with TAP-purified exosome, or box C/D snoRNP and the effect of Nop8p was analyzed. As previously shown, the purified exosome degrades poly-A RNA *in vitro*, whereas, box C/D snoRNP purified through the same procedure does not (Fig. 10A, lanes 5 and 9; [19]). Purified recombinant Nop53p or Nop8p, when incubated alone with poly-A RNA, did not degrade the substrate, similar to the negative control BSA (Fig. 10A, lanes 2–4). Interestingly, and contrary to Nop53p, that causes the exosome to degrade RNA more efficiently ([19]; Fig. 10A, lane 11), Nop8p inhibits the exosome RNase activity in a concentration dependent manner. These results indicate that Nop8p is an exosome interacting factor that can modulate its activity.

The RNA substrate used in these *in vitro* degradation assays was a single-stranded poly-A oligo, which has been shown to be efficiently degraded by the exosome [19]. To analyze whether the effect of Nop8p on the exosome activity was dependent on the substrate, we first compared the affinity of Nop8p towards poly-A and poly-AU RNA oligos. The results show that although Nop8p binds both RNA sequences, it has a much higher affinity for poly-AU sequences (Fig. S3). We next tested the effect of Nop8p on the exosome RNase activity using poly-AU as a substrate. The results show that the inhibitory effect of Nop8p upon the exosome activity is not dependent on the RNA sequence and therefore may not be dependent on Nop8p binding to the exosome substrate (Fig. 10B).

Because Rrp6p is one of the exosome catalytically active subunits, we decided to determine whether inhibition of RNA degradation by the exosome *in vitro* is at least partially due to Nop8p direct binding to Rrp6p. For this purpose, we next tested the effect of Nop8p on the activity of recombinant Rrp6p *in vitro* in the absence of the exosome complex. This assay showed that Nop8p inhibits Rrp6p activity (Fig. 10C, lanes 11–14) in a less efficient manner as compared to the inhibition observed in the assays with TAP-exosome. This may be due to conformational changes in the Rrp6p structure caused by protein-protein interactions when present in the exosome complex, or to additional changes caused by Nop8p and Rrp6p interaction with the exosome core.

## Discussion

In this work, we first mapped the Nop8p regions responsible for RNA and protein interactions. As shown here, the amino-terminal portion of Nop8p containing an RRM co-immunoprecipitates 5.8S rRNA, while the C-terminal portion of Nop8p interacts with Nip7p and partially complements the protein function. Another important finding is that Nip7p association to pre-ribosomal particles depends on the presence of full-length Nop8p, and that C-Nop8p causes the retention of Nip7p in these complexes. Based on the data obtained in this work, we can propose a model predicting that Nop8p binds to the pre-ribosomal particle before Nip7p and stabilizes the 27S pre-rRNA, possibly by preventing unspecific degradation by the exosome. According to our model, once Nop8p is bound to the complexes containing the 27S pre-rRNA, Nip7p can bind and thereby mediate cleavage of ITS2. This cleavage is probably carried out by the endonucleolytic activity of the exosome which so far has been the only nuclease associated with the excision of ITS2.

We also describe in this paper the interaction of Nop8p with the exosome subunit Rrp6p and inhibition of the exosome activity by recombinant Nop8p. The exosome is involved in processing and degradation of many types of RNAs and is a key component of the RNA-surveillance machinery in both the nucleus and the cytoplasm. The implication of the exosome in RNA processing in the nucleus and in RNA degradation in the nucleus and cytoplasm indicates that this complex interacts with cofactors specific for the different pathways. Indeed, Nop53p, Rrp47p, Mpp6p, and the TRAMP complex have been shown to act as nuclear exosome cofactors, whereas the Ski complex is a cytoplasmic exosome cofactor [19], [24]–[27]. It is interesting to note that many of the nuclear exosome cofactors already identified interact with Rrp6p, the subunit that is exclusively nuclear in yeast. Rrp47p is involved in processing of stable RNAs and also interacts with Rrp6p [28]. Mpp6p participates in the rapid degradation of nuclear ncRNAs and has been isolated in a synthetic lethality screen with *rrp6* mutants [25]. As shown here by protein pull-down assays, Nop8p interacts directly with Rrp6p. Depletion of Nop8p causes rRNAs degradation, probably through the quality control pathway involving the exosome. Nop53p participates in maturation of the 5.8S rRNA by directly interacting with Rrp6p and activating the exosome for processing of the 7S pre-rRNA [18], [19]. Here we show that Nop8p plays the opposite role of Nop53p, inhibiting the exosome activity *in vitro*. It will be interesting to determine the order of activity of each of these proteins during pre-rRNA processing and how their functions are controlled.

In addition to interacting with Nip7p [3], Nop8p has more recently been shown to interact genetically with the RNA helicase Dbp6p that participates in the early steps of assembly of the 60S particles, a process that requires many molecular rearrangements [4]. Nop8p has also been shown to be part of a protein complex, formed by Npa1p, Dbp6p, Has1p, and Rsa3p, that cosediments with pre-60S complex in polysomal gradients [10]. Interestingly, mutation in the *NPA1* and *NPA2* genes also leads to the degradation of 27S pre-rRNA and consequent decrease in the levels of the mature 25S and 5.8S rRNAs [9], [10], [20].

By involving the interaction between hundreds of factors, ribosome assembly is susceptible to errors in processing and folding of the pre-rRNA. Surveillance mechanisms are therefore crucial to eliminate defective precursors or processing intermediates, and the exosome plays an important role in nuclear rRNA quality control [29], [30]. Interestingly, in many cases, it is still not well understood how the correct and defective rRNA precursors can be distinguished from the correct ones by the surveillance machinery, but this process might involve auxiliary factors responsible for directing the exosome to its different substrates.

Depletion of Rrp6p causes the degradation of polyadenylated rRNAs from the 5′ to the 3′ direction [31]. In addition, mutants of the exosome subunits Rrp6p and Rrp46p have been shown to display accumulation of ITS2-containing 27S and 7S pre-rRNAs [32], [33]. Similarly, depletion of Nop8p leads to a strong decrease in the levels of mature 60S rRNAs. These results may indicate that the rRNA quality control machinery (of which the exosome is part) is more active in the absence of Nop8p, which would therefore control the activity of the complex by binding to target rRNAs and protecting them from degradation. Nop8p could inhibit the exosome through its direct interaction with Rrp6p. Alternatively, Nop8p could affect the exosome indirectly by binding to Nip7p, which also interacts with Rrp43p [3]. Our data obtained from *in vitro* RNA degradation favor the hypothesis that the exosome complex is inhibited by binding of Nop8p to the complex. Factors inhibiting the exosome activity have recently been described in archaea. *Pyrococcus abyssi* Nip7 was shown to interact with and strongly inhibit exosome activity *in vitro* [34]. As shown here, Nop8p is important for Nip7p binding to pre-ribosomes. It is therefore possible that Nop8p and Nip7p control the exosome activity during pre-rRNA processing. In the absence of Nop8p, the exosome degrades pre-rRNAs, causing a decrease in mature rRNAs. These results suggest that due to the more complex structure of the eukaryotic exosome and the larger variety of substrates, an increased number of protein factors is necessary for the control of eukaryotic exosome activity.

We mapped Nop8p regions responsible for RNA and protein interactions. The N-terminal portion of Nop8p containing an RRM co-immunoprecipitates the 5.8S rRNA, while the C-terminal portion of Nop8p contains a coiled-coil region, responsible for the interaction with Nip7p and probably also with Rrp6p. Accordingly, the C-terminal portion of Nop8p partially complements the protein function. Structural analysis of Rrp6p indicates that its catalytic domain plus the nucleic acid binding HRDC domain contract upon substrate binding. These two domains are connected by a linker region that is also part of a platform formed by the N-terminal domain [35]. This Rrp6p platform is involved in the interaction with Rrp47p, whereas the interaction with the core exosome involves Rrp6p C-terminal domain [28], [36]. Because the Nop8p interaction with Rrp6p causes the inhibition of Rrp6p RNase activity *in vitro*, it is tempting to speculate that Nop8p modulates Rrp6p function by also binding to its platform region.

The exosome has low activity *in vitro* [19], [26], suggesting a cellular defense mechanism against a broad-spectrum RNase. Activating factors may therefore be necessary *in vivo* for directing the exosome to its different substrates. Mtr4p, a DExH-box RNA helicase that is also a TRAMP subunit is involved in processing and degradation reactions catalyzed by the exosome [26], [37]. The TRAMP complex may activate the exosome by both interacting with it and by adding a poly-A tail to its targets, which creates a single-stranded 3′ stretch for the exosome to initiate degradation [16]. Pre-ribosome processing factors might in addition interact with the TRAMP and exosome complexes. Human Mpp6 and yeast Rrp47p and Nop53p are examples of such factors [19], [24], [38]. In an attempt to determine whether Nop8p could modulate TRAMP activity, we tested the interaction between these proteins through the two-hybrid system, but we detected only a weak interaction of Nop8p with Trf4p (data not shown).

Nop8p has 30% homology with the human protein NOP132 in the N-terminal region where they have RNA-binding motifs. Although the C-terminal regions of both proteins have only a 16% similarity, they have coiled-coil structure motifs, which are probably involved in protein interactions [39]. NOP132 downregulation caused mislocalization of DDX47, which led to the hypothesis that NOP132 recruits DDX47 to the nucleolus, where it functions in the processing of primary rRNA transcripts [6]. Similarly, depletion of yeast Nop8p causes the loss of Nip7p association with the pre-60S complex. Interestingly, depletion of Nop53p, a factor necessary for 5.8S rRNA formation, but with opposite effect on the exosome activity, also causes loss of Nip7p sedimentation with pre-60S complexes (Granato DC and Oliveira CC, unpublished results). These data indicate that Nip7p, a protein involved in later steps of 27S processing, binds only to correctly assembled pre-60S. The hypothesis that failure to successfully process 27S pre-rRNA hinders binding of later factors is supported by a model of ribosome assembly based on kinetic proofreading [16]. According to this model, some processing factors could recruit the exosome or other quality control factors when maturation is delayed, resulting in rRNA degradation. As shown here, in the absence of Nop8p, 60S pre-rRNAs are directed for degradation, in a process in which the exosome is a key player.

In summary, in this work we show the identification of an essential yeast protein involved in the control of the exosome function both *in vivo* and *in vitro*, what, to the best of our knowledge is the first example of a factor controlling the eukaryotic exosome by inhibition. This could shed light on the control mechanism that allows the release of mature and functional rRNAs by the exosome during processing, whereas in the quality control pathway, exosome substrates get completely degraded.

## Materials and Methods

### Plasmids construction

The plasmids used in this study, described in Table 1, were constructed according to the cloning techniques described in [40], and sequenced by the Big Dye method (PerkinElmer, Waltham, MA, USA). For the construction of pET-NOP8 and pGEX-NOP8, the *NOP8* DNA fragment was obtained from pGFP-NOP8 vector [3] digested with BamHI and XhoI and inserted into the pET-28a (Merck KGaA, Darmstadt, Germany) and pGEX-4T1 (GE Healthcare, Little Chalfont, UK) vectors digested with the same enzymes. To obtain the Nop8p truncation mutants, plasmid pGFP-NOP8 [3] was cleaved with BamHI, HincII and XhoI, resulting in two fragments coding for Nop8p amino acid residues 1–219 (containing the RRM motif) and residues 221–484 (containing the coiled-coil and unstructured regions), which were cloned into pGADC1 [41] and pBTM116 [42], generating pBTM- and pGAD–N-NOP8 (1–219) and pBTM- and pGAD-C-NOP8 (221–484). *NOP8* DNA fragments coding for the N-terminal (amino acids 1–219) and C-terminal (amino acids 221–484) portions of the protein were PCR amplified from plasmid pGFP-NOP8, digested with BamHI and XhoI and cloned into vectors pUG34 (U. Gueldener & J. H. Hegemann, unpublished, under the control of MET25 promoter) pGEXT-4T1 and YCplac33GAL-A, fused to the protein A tag, under the control of the *GAL1* promoter.

**Table 1:** List of plasmid vectors used.

Plasmid	Relevant characteristics	Reference
pBTM116	*lexA* DNA binding domain, *TRP1*, 2 µm	42
pBTM-NOP8	*lexA::NOP8*, *TRP1*, 2 µm	3
pBTM-N-NOP8	*lexA::N-NOP8*, *TRP1*, 2 µm	This study
pBTM-C-NOP8	*lexA::C-NOP8*, *TRP1*, 2 µm	This study
pGAD	*GAL4* activation domain, *LEU2*, 2 µm	41
pACT-NOP8	*GAL4::NOP8*, *LEU2*, 2 µm	3
pACT-N-NOP8	*GAL4::N-NOP8*, *LEU2*, 2 µm	This study
pACT-C-NOP8	*GAL4::C-NOP8*, *LEU2*, 2 µm	This study
pET28a-NOP8	*His6::NOP8*, *KanR*	This study
pGEX4T1-NOP8	*GST::NOP8*, *AmpR*	This study
pGEX4T1-N-NOP8	*GST::N-NOP8*, *AmpR*	This study
pGEX4T1-C-NOP8	*GST::C-NOP8*, *AmpR*	This study
pUG34-NOP8	*MET*25*::yEGFP3-NOP8*, *HIS3*, *CEN6*	This study
pUG34-N-NOP8	*MET*25*::yEGFP3-N-NOP8*, *HIS3*, *CEN6*	This study
pUG34-C-NOP8	*MET*25*::yEGFP3-C-NOP8*, *HIS3*, *CEN6*	This study
YCp33GAL-A	*GAL1::PROTA*, *URA3*, *CEN4*	18
YCp33GAL-A-NOP8	*GAL1::PROTA-NOP8*, *URA3*, *CEN4*	This study
YCp33GAL-A-N-NOP8	*GAL1::PROTA-N-NOP8*, *URA3*, *CEN4*	This study
YCp33GAL-A-C-NOP8	*GAL1::PROTA-C-NOP8*, *URA3*, *CEN4*	This study
pET28a-NIP7	*His6::NIP7*, *KanR*	43
pET28a-NOP53	*His6::NOP53*, *KanR*	18
pET28a-NOP17	*His6::NOP17*, *KanR*	44
pGEX4T1-RRP6	*GST::RRP6*, *AmpR*	19
pGEX4T1-TRF4	*GST::TRF4*, *AmpR*	19
pUG34	*MET*25*::yEGFP3*, *CEN6*, *HIS3*	U. Gueldener and J. H. Hegemann, unpublished

### Maintenance and handling of *E. coli* and yeast strain

*Escherichia coli* strains DH5α and BL21(DE3) were maintained in LB medium and manipulated according to standard techniques [40]. The yeast strains used in this work, with a brief description of the relevant genetic markers, are shown in Table 2. The yeast strains of *S. cerevisiae* were maintained in yeast extract–peptone (YP) medium or synthetic medium (YPD) with 2% (w/v) galactose or glucose as the carbon source, as indicated, and supplemented with amino acids when required. Yeast cells were transformed using a lithium acetate method [46]. Carbon source-conditional strains were incubated in YP medium containing 2% galactose, and transferred to 2% glucose for the indicated periods of time. For the growth curve in liquid medium, cells were grown in medium containing galactose until stationary phase and then shifted to glucose medium for 24 h.

**Table 2:** Yeast and bacteria strains used in this study.

Strain	Relevant characteristics	Reference
DG 456	MAT**a** ade2-1 leu2-3,112 his3-11,15 trp1-1 ura3-1 can1-100 Nop8::KAN p(URA3 GAL::PrtA::NOP8)	3
YMS 52	*W303*, YCp33GAL-A	This study
YMS 53	*W303*, YCp33GAL-A-NOP8	This study
YMS 54	*W303*, YCp33GAL-A-N-NOP8	This study
YMS 55	*W303*, YCp33GAL-A-C-NOP8	This study
YMS 20	*Δnop8*, YCp33GAL-A-NOP8,pACT-NOP8	This study
YMS 21	*Δnop8*,YCp33GAL-A-NOP8,pGAD-N-NOP8	This study
YMS 22	*Δnop8*,YCp33GAL-A-NOP8,pGAD-C-NOP8	This study
YMS 13	*Δnop8*, YCp33GAL-A-NOP8,pGADC_1_	This study
YMS 43	*Δnop8, YCp33GAL-A-NOP8,GFP-NOP8*	This study
YMS 44	*Δnop8,YCp33GAL-A-NOP8,GFP-N-NOP8*	This study
YMS 45	*Δnop8, YCp33GAL-A-NOP8,GFP-C-NOP8*	This study
YMS 46	*Δnop8, YCp33GAL-A-NOP8,GFP*	This study
DH5α	*supE*44 *Δlac*U169 *(ϕ80 lacZΔ*M*15) hsdR17 rec*A1 *endA1 gyrA96 thi1 relA1*	45
BL21 Codon Plus (DE3) RIL	*E. coli* B F– ompT hsdS(r_B_– m_B_–) dcm+ Tet^r^ gal l (DE3) endA Hte [argU ileY leuW Cam^r^]*	Stratagene

### Yeast two-hybrid assays

Fusion proteins with either *lex*A DNA-binding domain (BD-protein) or Gal4p transcription activation domain (AD-protein) were expressed in the host strain L40 [47], which has two reporter genes for two-hybrid interactions integrated into the genome: yeast *HIS3* and *E. coli lac*Z. Transformants were plated in minimal medium lacking histidine for first selection and the viable clones were further tested for β-galactosidase activity as follows. Exponentially growing cultures in minimal medium (supplemented with histidine) were concentrated 10-fold and either transferred to nitrocellulose membranes and incubated overnight at 30°C for the β-galactosidase activity assay [47], or plated in medium lacking histidine.

### RNA analysis

Exponentially growing cultures of yeast strains were shifted from galactose to glucose medium. At various times, samples were collected and quickly frozen. Total RNA was isolated from yeast cells by a modified hot phenol method [48]. RNAs were separated by electrophoresis on 1.3% agarose gels, following denaturation with glyoxal and transferred to Hybond nylon membranes (GE Healthcare). Membranes were probed with ^32^P-labeled oligonucleotides complementary to specific regions of the 35S pre-rRNA, using the hybridization conditions described previously [49] and analyzed in a Phosphorimager (Molecular Dynamics, Sunnyvale, CA, USA).

### In vitro RNA degradation assay

Assay was performed as described previously [19]. Radioactively labeled poly-A_14_ or poly-AU_21_ RNA oligos were incubated with 2 µM of the exosome, or 5 µM of box C/D snoRNP, and recombinant His-Nop8p, His-Nop53p or BSA. Reactions were incubated at 37°C for 30 minutes and analyzed by denaturing polyacrylamide gel electrophoresis.

### Quantitative analysis of RNA by qPCR

Total RNA was extracted from cultures incubated in triplicates. 1 µg of total RNA was reverse-transcribed using the ThermoScript™ RT-PCR System (Invitrogen, Carlsbad, CA, U.S.A) with 50 µM of oligo(dT)_20_ or 50 ng of random hexamers primers in a 20 µl reaction. The reverse transcriptase product was used as template in qPCR reactions with Maxima SYBR Green/ROX qPCR Master Mix (Molecular Probes Inc., Eugene, OR, U.S.A.). The data are presented as the fold change in gene expression normalized to an unaffected reference gene, the scR1 RNA, and are relative to the time zero sample. For time zero sample, ΔΔC^T^ is zero, and 2^−ΔΔC^_T_ = 2^0^ equals one. Therefore the fold change in gene expression relative to the time zero is one. For each time point sample, evaluation of 2^−ΔΔC^_T_ indicates the fold change in gene expression relative to the time zero control [50]. Primers are described in Table 3.

**Table 3:** Oligonucleotides used for Northern blot hybridization or qPCR.

Oligo	Sequence	Reference
18S	5′-CATGGCTTAATCTTTGAGAC-3′	51
5.8S	5′-CGTATCGCATTTCGCTGCGTTC-3′	49
DE2	5′-CTCACTACCAAACAGAATGTTTGAGAAGG-3′	15
UC1	5′-GTTCGCCTAGACGCTCTCTTC-3′	49
25S	5′-GCCGCTTCACTCGCCGTTACTAAGGC-3′	44
5S	5′-GGTCACCCACTACACTACTCGG-3′	18
Scr1 Rev	5′-TCTAGCCGCGAGGAAGGA-3′	52
5.8SqPCRFor	5′-GCGAAATGCGATACGTAATGTG-3′	This study
5.8SqPCRRev	5′-GGCGCAATGTGCGTTCA-3′	This study
18SqPCRFor	5′-GTGCATGGCCGTTCTTAGTTG-3′	This study
18SqPCRRev	5′-AGGTTAAGGTCTCGTTCGTTATCG-3′	This study
25SqPCRFor	5′-CCGGGATTGCCTTAGTAACG-3′	This study
25SqPCRRev	5′-GGCACCGAAGGTACCAGATTT-3′	This study
ITS2qPCRFor	5′-TTTCTCTGCGTGCTTGAGGTATAA-3′	This study
ITS2qPCRRev	5′-AAAAGATTAGCCGCAGTTGGTAA-3′	This study
5SqPCRFor	5′-TTTCCCGTCCGATCAACTG-3′	This study
5SqPCRRev	5′-GCGTATGGTCACCCACTACACTAC-3′	This study
Scr1qPCRFor	5′-TCCTTCCTCGCGGCTAGA-3′	This study
Scr1qPCRRev	5′-GCACGGTGCGGAATAGAGA-3′	This study

### Co-immunoprecipitation of RNAs

Total cellular extracts were prepared from yeast strains expressing the ProtA, ProtA-Nop8p, ProtA-N-Nop8p or ProtA-C-Nop8p, and added to IgG-Sepharose beads (GE Healthcare, Little Chalfont, UK). Immunoprecipitation was performed at 4°C for 2 h. IgG-Sepharose beads were washed with buffer containing 10 mM Tris-Cl pH 7.4, 5 mM magnesium chloride, 0.1% Nonidet P-40, 150 mM sodium chloride, 1 mM DTT and protease inhibitors. The RNA was isolated from bound fractions by adding phenol directly to the beads. After precipitation, the recovered RNA was used as template in reverse transcriptase reactions.

### Primer extension analysis

Total RNA extracted as described above was used for primer extension analysis. Reactions were performed by annealing 1 pmol of ^32^P-labeled oligonucleotide to 5 µg of total RNA. Following annealing, extension was performed with 100 U of Moloney's Murine Leukemia Virus (MMLV) reverse transcriptase (Invitrogen, Carlsbad, CA, USA) and dNTPs (0.5 mM) for 30 min at 37°C. The cDNA products were precipitated, suspended in H_2_O, treated with RNase A, denatured and analyzed on 6% denaturing polyacrylamide gels. Gels were dried and analyzed in a Phosphorimager (Molecular Dynamics, Sunnyvale, CA, USA). Oligonucleotides used in primer extension analyses are listed in Table 3.

### Polysome profile analysis

For polysome profile analysis cell extracts from 500 ml cultures grown to *A*_600_ 1.0 in YNB-Gal (*t*_0_) or in YNB-Glu for 6 or 15 hours were used. Following addition of cycloheximide (100 µg/ml) to the cultures, cells were harvested by centrifugation, suspended in breaking buffer A (20 mM Tris/HCl pH 7.4, 50 mM NaCl, 10 mM MgCl_2_, 1 mM DTT, 200 µg/ml heparin, 100 µg/ml cycloheximide, 1 mM PMSF) and lysed using glass beads. Polysomes were separated by centrifugation at 190,000 ***g*** for 3 h at 4°C with a Beckman SW41 rotor. Gradients were fractionated and monitored at 254 nm with an absorbance monitor (BioRad). Analysis of free ribosome subunits was performed as described above, using breaking buffer B (20 mM Tris/HCl pH 7.4, 50 mM NaCl, 400 mM EDTA, 1 mM PMDF, 1 mM DTT, 200 µg/ml heparin, 100 µg/ml cycloheximide). Proteins from each fraction (500 µl) were precipitated with 15% trichloroacetic acid and analyzed by western blot with specific antibodies. For northern blot experiments, 1.5 ml of cold ethanol was added to 500 µl of each fraction. Pellets were suspended in 500 µl of acetate buffer (50 mM NaOAc, 10 mM EDTA, pH 5.0) and RNA was isolated as described above.

### Protein pull-down and immunoblot

For pull-down of His-Nop8p, whole-cell extracts from *E. coli* cells expressing either GST or GST-Rrp6p were generated in buffer containing 50 mM Tris/HCl pH 8.0, 150 mM sodium chloride, 0.2% v/v Triton X-100, 1 mM PMSF and mixed with 500 µl of glutathione–sepharose beads (GE Healthcare, Little Chalfont, UK). After washing bound material with the same buffer, whole-cell extracts from *E. coli* cells expressing His-Nop8p were added to the glutathione-sepharose beads and incubated at 4°C for 2 h. The glutathione-sepharose beads were precipitated and washed again with the initial buffer, and bound proteins were eluted and resolved on SDS-PAGE, and transferred to poly(vinylidene) difluoride (PVDF) membranes (GE Healthcare), which were incubated with anti-(poly histidine) antibody (GE Healthcare, Little Chalfont, UK) or anti-GST serum (Sigma, St Louis, MO, USA). The immunoblots were developed using the Immobilon™ Western Chemiluminescent HRP Substrate (Millipore Corporation, Billerica, MA, U.S.A).

### Immunoprecipitation of complexes by TAP-Tag methodology

The purification of complexes using TAP-Rrp43p and TAP-Nop58p was performed as described previously [19]. Yeast cells expressing TAP-Rrp43p or TAP-Nop58p were grown in 2 l of YPD medium. Isolation of the complexes was performed by incubating yeast total extracts for 2 h at 4°C with IgG-Sepharose beads (GE Healthcare, Little Chalfont, UK), followed by extensive washing with TMN buffer containing 10 mM Tris pH 7.6, 100 mM sodium chloride, 5 mM magnesium chloride, 0.1% Nonidet P-40, 1 mM DTT, 1 mM PMSF. The exosome and box C/D snoRNP complexes were eluted from the beads by incubating the resin with 20 U of Tobacco Etch Virus protease (Invitrogen, Carlsbad, CA, U.S.A) for 16 h at 4 C.

## Supporting Information

Figure S1 **Fractionation of ribosomal subunits through sucrose gradient.** Analysis of the ribosomal subunits levels in strain *Δnop8/GAL1::NOP8* compared to the same strain expressing either Nop8p, N-Nop8p or C-Nop8p, incubated in glucose medium for 15 hours. Upper left panel, *Δnop8/GAL1::NOP8* strain. Upper right panel, *Δnop8/GAL1::NOP8/NOP8* strain. Lower left panel, *Δnop8/GAL1::NOP8/N-NOP8*. Lower right panel, *Δnop8/GAL1::NOP8/C-NOP8*. Expression of C-Nop8p is sufficient for restoring 60S subunit levels.
(TIF)

Figure S2 (**A**–**B**) Analysis of protein complexes recovered through TAP purification. TAP-Rrp43p co-purified Mtr3p (**A**) and TAP-Nop58p co-purified Nop1p (**B**), indicating that the exosome and box C/D snoRNP complexes, respectively, were intact. (**C**) Silver staining of purified proteins used in *in vitro* RNase activity assays.
(TIF)

Figure S3 **Analysis of Nop8p interaction with RNA oligonucleotides** ***in vitro*** **.** Electrophoretic mobility shift assays with radiolabeled RNA probes incubated with the indicated amounts of purified proteins. Proteins were incubated with 1 pmol of 14-mer poly-rA, or 21-mer poly-rAU RNA oligos at 37°C for 30 min. RNA-protein complexes were fractionated on 8% native polyacrylamide gels and visualized by phosphorimaging. –, No protein was added to the reaction. Free RNA oligos and protein-RNA complexes are indicated on the right hand side.
(TIF)
