# Supplementary figures and images for: The Essential Nucleolar Yeast Protein Nop8p Controls the Exosome Function during 60S Ribosomal Subunit Maturation
Source: PLoS One. 2011 Jun 29;6(6):e21686. doi: 10.1371/journal.pone.0021686 (PMC3126838; doi:10.1371/journal.pone.0021686)

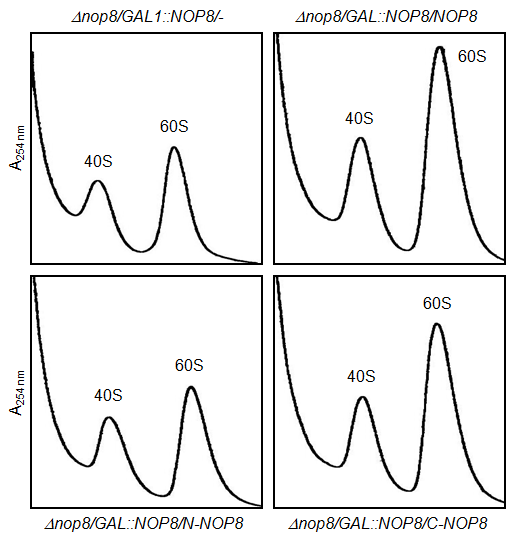

Supplement: Figure S1 — Fractionation of ribosomal subunits through sucrose gradient. Analysis of the ribosomal subunits levels in strain Δnop8/GAL1::NOP8 compared to the same strain expressing either Nop8p, N-Nop8p or C-Nop8p, incubated in glucose medium for 15 hours. Upper left panel, Δnop8/GAL1::NOP8 strain. Upper right panel, Δnop8/GAL1::NOP8/NOP8 strain. Lower left panel, Δnop8/GAL1::NOP8/N-NOP8. Lower right panel, Δnop8/GAL1::NOP8/C-NOP8. Expression of C-Nop8p is sufficient for restoring 60S subunit levels. (TIF) [file pone.0021686.s001.tif]

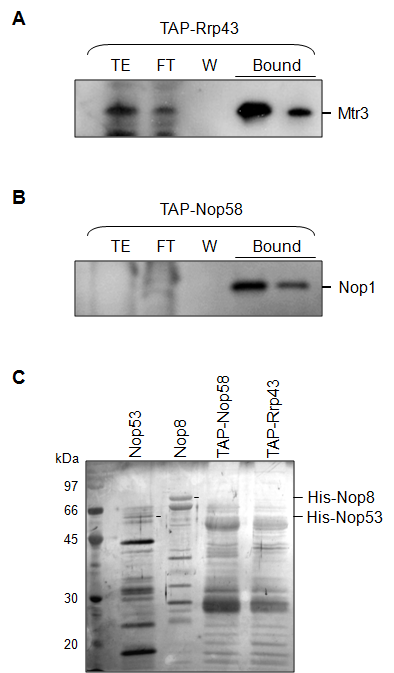

Supplement: Figure S2 — (A–B) Analysis of protein complexes recovered through TAP purification. TAP-Rrp43p co-purified Mtr3p (A) and TAP-Nop58p co-purified Nop1p (B), indicating that the exosome and box C/D snoRNP complexes, respectively, were intact. (C) Silver staining of purified proteins used in in vitro RNase activity assays. (TIF) [file pone.0021686.s002.tif]

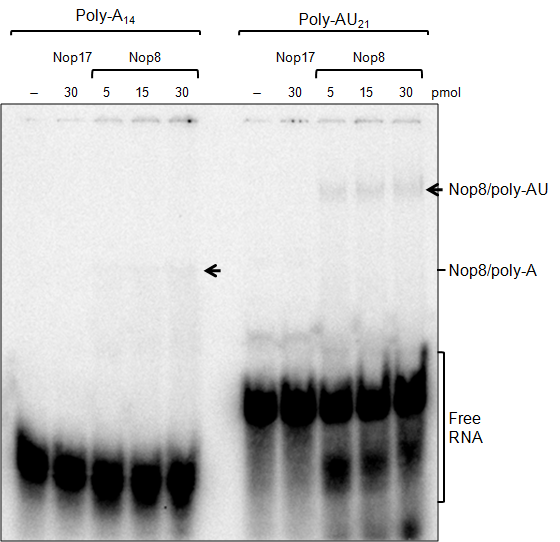

Supplement: Figure S3 — Analysis of Nop8p interaction with RNA oligonucleotides in vitro . Electrophoretic mobility shift assays with radiolabeled RNA probes incubated with the indicated amounts of purified proteins. Proteins were incubated with 1 pmol of 14-mer poly-rA, or 21-mer poly-rAU RNA oligos at 37°C for 30 min. RNA-protein complexes were fractionated on 8% native polyacrylamide gels and visualized by phosphorimaging. –, No protein was added to the reaction. Free RNA oligos and protein-RNA complexes are indicated on the right hand side. (TIF) [file pone.0021686.s003.tif]
